# Mechanisms of Ibuprofen Retention and Release in Dual-Responsive P(NIPAM-co-AAc) Nanogels: Coupling of Mesh Sieving and Affinity Switching

**DOI:** 10.3390/gels12050379

**Published:** 2026-04-30

**Authors:** Yiqi Zhou, Haodong Yao, Bicheng Han, Jihong Sun, Huijie Ge, Shiyang Bai, Lina Zhao

**Affiliations:** 1 Beijing Key Laboratory for Green Catalysis and Separation, Institute of Matter Science, Beijing University of Technology, Beijing 100124, China; 2 Multi-Disciplinary Research Division, Institute of High Energy Physics, Chinese Academy of Sciences, Beijing 100049, China; 3 State Key Laboratory of High Performance Ceramics, Shanghai Institute of Ceramics, Chinese Academy of Sciences, Shanghai 200050, China

**Keywords:** P(NIPAM-co-AAc) nanogels, multi-stimuli-responsive drug delivery, molecular dynamics simulation, mesh size, hydrogen bond

## Abstract

Rational design of smart nanogels for drug delivery requires molecular-level understanding of how structural evolution and drug–carrier interactions couple under multiple stimuli. Here, pH/temperature dual-responsive P(NIPAM-co-AAc) nanogels containing 0–20 mol% AAc were investigated by combining all-atom molecular dynamics simulations with in vitro ibuprofen (IBU) release experiments under acidic (pH 2.75) and weakly basic (pH 7.4) conditions at 298 and 310 K. The simulations identified CA-5-L-298 as the most retained system, with the lowest IBU diffusion coefficient (0.92 × 10^−7^ cm^2^ s^−1^) and no dissociated molecules under the adopted criterion, whereas CA-15-H-310 showed the highest diffusivity (8.61 × 10^−7^ cm^2^ s^−1^) and dissociated fraction (22%). Consistently, in the urea-free release experiments, CA-15-H-310 exhibited the highest 24 h cumulative release (69.4%), while CA-5-L-298 remained among the low-release systems (35.9%). Pore analysis, hydrogen-bond statistics, MM/PBSA calculations, and urea-competition experiments together support the view that IBU release is influenced by both mesh steric sieving and polymer–drug affinity switching, and correlation analysis provides quantitative support for linking the MD descriptors with the experimental release behavior. Overall, the simulations reproduce the qualitative trends in the experiments and provide a molecular-level framework for rationalizing the observed release behavior in dual-responsive nanogels.

## 1. Introduction

Multi-stimuli-responsive nanogels are attractive drug carriers because they can couple environmental sensing with changes in network hydration, conformation, and transport behavior [1,2,3,4,5]. Among these systems, poly(N-isopropylacrylamide) (PNIPAM)-based systems are particularly important because PNIPAM exhibits a physiologically relevant lower critical solution temperature (LCST), while copolymerization with acrylic acid (AAc) introduces pH-dependent ionization and thus enables dual pH/temperature responsiveness [6,7,8]. For P(NIPAM-co-AAc) carriers, previous experimental studies have shown that ibuprofen (IBU) loading and release depend strongly on pH, temperature, and copolymer composition. In particular, increasing the AAc content can alter swelling, aggregation, and microstructural features of the carrier, leading to marked differences in release behavior [8,9,10,11]. Taken together, prior experimental studies on P(NIPAM-co-AAc) carriers indicate that drug loading and release depend strongly on pH, temperature, and copolymer composition. They also suggest that variations in AAc content modify swelling, aggregation, and microstructural heterogeneity, which in turn alter release behavior. However, these conclusions have largely been drawn from bulk swelling, particle-size, and dried-state structural descriptors [12], rather than from a direct molecular picture of transport through the hydrated network [9,10,13,14,15,16,17].

Molecular simulation provides a complementary route to resolve this problem at the molecular scale. All-atom molecular dynamics (MD) simulations have been used to quantify drug–polymer interactions and aggregation tendencies in drug-delivery formulations, showing that molecular affinity can strongly influence drug mobility and stability [18]. In pH-responsive carrier systems, simulation studies further showed that carrier aggregation and carrier–drug interactions can act as competing controls on drug release [19]. More recently, MD has been extended to chemically cross-linked PNIPAM networks to examine temperature-sensitive behavior and ibuprofen release [20]. Nevertheless, the current simulation literature has focused mainly on PNIPAM homopolymer systems or single-stimulus cases, and has not yet resolved how AAc content and pH-dependent ionization jointly reshape hydrated pore accessibility and polymer–drug affinity in dual-responsive, explicitly cross-linked P(NIPAM-co-AAc) nanogels [21].

Accordingly, this work aims to provide a mechanistic interpretation of IBU retention and release in a known dual-responsive nanogel platform. Here, we combine all-atom molecular dynamics (MD) simulations with in vitro release experiments to investigate explicitly cross-linked poly(N-isopropylacrylamide-co-acrylic acid) [P(NIPAM-co-AAc)] nanogels with varying AAc contents under acidic and weakly basic conditions at 298 and 310 K. Rather than treating release as a simple consequence of bulk swelling, we examine a coupled mechanism in which hydrated pore accessibility governs whether ibuprofen (IBU) can traverse the network, while polymer–drug affinity determines whether accessible pathways translate into actual release. By comparing pore limiting diameter (PLD) and maximum pore diameter (MPD) with the molecular dimensions of IBU, and by integrating molecular mechanics/Poisson–Boltzmann surface area (MM/PBSA) analysis, hydrogen-bond classification, and urea-competition release experiments, this work establishes a clearer structure–mechanism–performance picture for IBU retention and release in dual-responsive P(NIPAM-co-AAc) nanogels.

## 2. Results and Discussion

### 2.1. Dual pH/Temperature Regulation of Ibuprofen Diffusion and Release

The diffusion coefficients (*D*) of IBU and the corresponding dissociated fraction for all systems are summarized in Table 1. Comparison of the diffusion coefficients shows that CA-5-L-298 exhibited the lowest *D* value (0.92 × 10^−7^ cm^2^ s^−1^), corresponding to an IBU dissociated fraction of 0%. In contrast, CA-15-H-310 displayed the highest *D* value (8.61 × 10^−7^ cm^2^ s^−1^), and its dissociated fraction reached 22%. Therefore, these two systems were selected as representative cases for detailed comparison. After an initial conformational relaxation, the root-mean-square deviation (RMSD) curves of all systems reached stable plateaus (Figures S1 and S2), indicating that the subsequent diffusion and structural analyses were performed on equilibrated trajectories. In general, the low-pH systems equilibrated somewhat earlier and exhibited smaller late-stage fluctuations than the corresponding high-pH systems. The MSD curves and the corresponding linear fitting regions used to obtain the diffusion coefficients are provided in Figures S4 and S5, further supporting the differences in IBU diffusional behavior discussed here.

**Figure 1:**
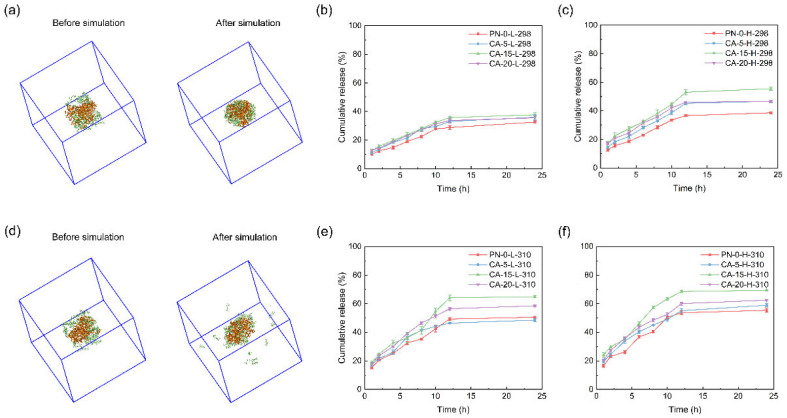
Snapshots before (**left**) and after (**right**) simulation for two IBU-loaded nanoparticles: (**a**) CA-5-L-298 and (**d**) CA-15-H-310. In the molecular dynamics snapshots, the nanoparticles are shown in orange representation, and IBU molecules are shown in lime CPK representation. (**b**,**c**,**e**,**f**) Cumulative IBU release profiles from the nanoparticles in release medium under different temperature–pH conditions: (**b**) 298 K, pH 2.75; (**c**) 298 K, pH 7.4; (**e**) 310 K, pH 2.75; (**f**) 310 K, pH 7.4. In each panel, curves correspond to PN-0, CA-5, CA-15, and CA-20 nanoparticles. Cumulative IBU release (%) data are presented as mean ± SD (*n* = 3 independent experiments). Error bars represent SD.

Figure 1 shows that the simulation trends are qualitatively consistent with the experimental release profiles, with CA-5-L-298 and CA-15-H-310 selected as representative retained and release-favored cases.

In CA-5-L-298, IBU molecules progressively enriched near the nanoparticle and remained closely associated with the carrier in the final snapshot. This retention is consistent with the suppressed cumulative in vitro release, which reached only 35.9% at 24 h. Consequently, the desorption and the outward redistribution of the drug away from the carrier are hindered. This qualitative agreement between molecular configuration evolution and macroscopic release behavior suggests that the simulations capture the qualitative trends and help rationalize the observed release behavior.

In contrast, for the CA-15-H-310 system shown in Figure 1d, a substantial fraction of IBU molecules exhibited a tendency for outward diffusion, with some even detaching from the nanoparticle surface. This aligns with the experimental results in Figure 1f, which reached the highest 24 h cumulative release (69.4%) among the urea-free systems.

To characterize the release-relevant dissociation behavior of IBU from the nanogel, the average minimum distance between each of the 50 IBU molecules and the nanoparticle was calculated over the final 10 ns. A distance of 5 Å was adopted as an operational criterion for direct contact, as it encompasses the typical range of short-range intermolecular interactions (e.g., van der Waals contacts and hydrogen bonds). To quantify release-relevant dissociation, an IBU molecule was classified as dissociated when its average minimum distance from the polymer exceeded 5 Å over the final 10 ns, indicating the loss of direct short-range contact [22,23]. The resulting IBU dissociated fractions are summarized in Table 1 and defined as follows:

$$f_{det}\left(\%\right)=\frac{N_{det}}{N_{IBU}}\times 100\%$$

where $N_{det}$ is the number of IBU molecules classified as dissociated, and $N_{IBU}$ is the total number of IBU molecules.

Based on this criterion, the PN-0 systems retained measurable dissociated fractions under all conditions, whereas the copolymer systems showed negligible dissociation under acidic conditions at 298 K. Under weakly basic conditions at 310 K, the dissociated fraction increased up to CA-15 and then decreased for CA-20, suggesting that stronger release does not increase monotonically with AAc content.

The release behavior was evaluated using several model-dependent kinetic equations, including the zero-order, first-order, Higuchi, and Korsmeyer–Peppas models [24]. The fitting results for all formulations are summarized in Table 2. Among these models, the Korsmeyer–Peppas model showed the best goodness of fit for all systems, with the highest *R*^2^ values (0.931–1.000), compared with the first-order (0.880–0.951), Higuchi (0.698–0.894), and zero-order models, which showed the poorest fit. In addition, the release exponent *n* obtained from the Korsmeyer–Peppas model ranged from 0.256 to 0.408. Since all *n* values were below 0.5, the drug release mechanism could be considered to follow Fickian diffusion.

### 2.2. Spatial Distribution of Ibuprofen in the Nanogels

**Figure 2:**
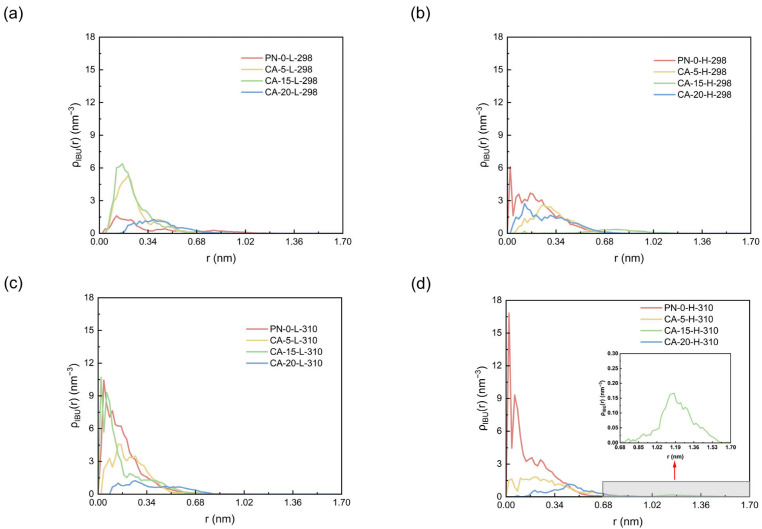
Radial number density profiles, ρ(r), of IBU relative to the polymer center of mass for all simulated systems under four modeled conditions: (**a**) 298 K, pH 2.75; (**b**) 298 K, pH 7.4; (**c**) 310 K, pH 2.75; and (**d**) 310 K, pH 7.4.

To further characterize the spatial distribution of IBU within the nanogels, we calculated the radial number density profiles, ρ(r), of IBU relative to the polymer center of mass, as shown in Figure 2a–d. Representative final configurations for all simulated systems are compiled in Figure S3, providing visual support for the condition- and composition-dependent differences in IBU distribution discussed below. A higher density at smaller r indicates that IBU is preferentially distributed at small r, whereas a broader distribution shifted toward larger r reflects outward redistribution of the drug molecules and weaker retention.

Under acidic conditions at 298 K, CA-5 and CA-15 show pronounced enrichment in the small-r region, whereas CA-20 exhibits a more dispersed distribution.

Under weakly basic conditions at 298 K (Figure 2b), the radial number density profiles become broader overall and shift moderately toward larger r, indicating partial outward migration of IBU from the nanogel interior. Among these copolymer systems, CA-15-H-298 exhibits the most pronounced outward displacement, consistent with its larger dissociated fraction relative to the corresponding acidic system. This trend suggests that deprotonation weakens the local retention of IBU and promotes its redistribution from smaller to larger r.

Under acidic conditions at 310 K (Figure 2c), CA-15-L-310 shows the strongest enrichment in the small-r region, CA-5-L-310 remains enriched but more dispersed, and CA-20-L-310 shows the weakest enrichment. These results indicate a clear composition dependence of IBU localization under these conditions.

Under weakly basic conditions at 310 K (Figure 2d), all systems show weaker local retention and greater outward redistribution. CA-15-H-310 extends furthest toward larger r, whereas the outward shift in CA-20-H-310 remains less pronounced. This finding indicates that weak polymer–drug affinity alone is insufficient to ensure a high dissociated fraction. It also suggests that a more diffuse spatial distribution does not necessarily correspond to a higher dissociated fraction, because further outward migration of the drug may still be constrained by the network mesh size.

It should also be noted that the limited differences observed for PN-0 between different pH conditions arise primarily not from the intrinsic pH response of the carrier itself, but from changes in the ionization state of IBU. Under acidic conditions, neutral IBU is less hydrated and therefore more readily retained near PNIPAM through van der Waals/hydrophobic contacts and hydrogen bonding. Under weakly basic conditions, deprotonated IBU becomes more strongly solvated and less compatible with the relatively hydrophobic PNIPAM microenvironment, thereby showing a greater tendency to redistribute toward larger radial distances and more readily lose direct contact with the polymer. This interpretation is also consistent with the trend in Table 1 showing that polymer–IBU hydrogen bonds decrease to nearly zero under high-pH conditions.

### 2.3. Interactions Between Nanoparticles and Ibuprofen

**Figure 3:**
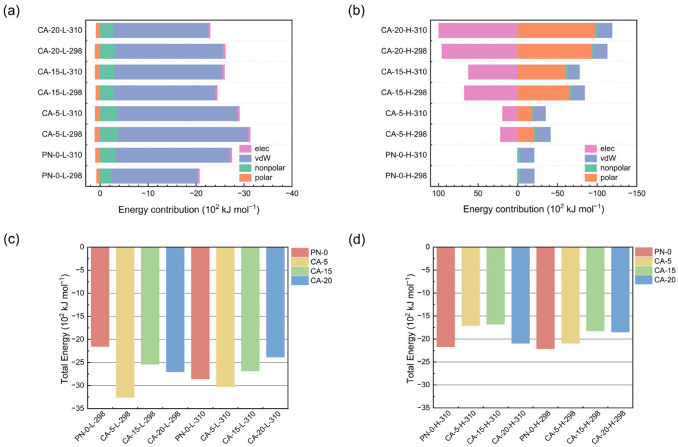
Decomposed and total interaction energies between IBU and nanoparticle. (**a**,**b**) Decomposition of the interaction into elec, vdW, nonpolar and polar contributions. Total binding energies at (**c**) pH 2.75 and (**d**) pH 7.4.

The release-relevant dissociation and transport behavior is closely associated with the interaction energy between the drug and the carrier. To quantify this, binding free energies were calculated using the MM/PBSA approach as implemented in gmx_MMPBSA, which interfaces GROMACS trajectories with AmberTools [25,26]. The decomposed interaction terms are summarized in Figure 3 with the corresponding totals listed in Table S3. In the present systems, pH acts as a key control parameter: at pH 2.75, IBU and AAc units are predominantly protonated, whereas at pH 7.4, both are mainly deprotonated, which markedly alters the balance between attractive and repulsive drug–polymer interactions. For PNIPAM-based copolymers containing AAc, the resulting response is commonly interpreted as a competition between PNIPAM-driven hydrophobic association/collapse and charge-induced electrostatic repulsion plus osmotic hydration arising from ionized carboxyl groups.

As shown in Figure 3a, all systems at pH 2.75 exhibit the same overall energetic pattern: the vdW term is the dominant favorable contribution, the nonpolar term is also favorable, the polar term is unfavorable, and the elec contribution remains small. This decomposition indicates that, when both the drug and the AAc units are largely uncharged, IBU retention is governed mainly by short-range noncovalent contacts rather than by Coulombic attraction. Among all nanoparticles, CA-5 shows the most favorable total binding free energy at this pH, reaching −30.198 × 10^2^ kJ mol^−1^ at 298 K and −28.033 × 10^2^ kJ mol^−1^ at 310 K (Figure 3c, Table S3). Relative to PN-0-L-298, the stronger binding in CA-5-L-298 arises primarily from a more favorable vdW contribution together with a more favorable nonpolar term, showing that moderate AAc incorporation can strengthen local drug–polymer contact without eliminating the nonpolar interaction environment provided by PNIPAM segments. This interpretation is consistent with earlier experimental work on P(NIPAM-co-AA) carriers showing that introducing acrylic acid enhances IBU loading while maintaining pronounced pH/temperature responsiveness [9].

A markedly different interaction pattern appears at pH 7.4 (Figure 3b). Under this condition, the carboxyl groups of both AAc and IBU are predominantly deprotonated, resulting in stronger electrostatic repulsion between the negatively charged polymer segments and IBU in the AAc-containing systems and thus favoring IBU dissociation from the nanoparticles. This is reflected by the large positive elec terms, which increase sharply with AAc content, from 21.859 × 10^2^ kJ mol^−1^ for CA-5-H-298 to 95.643 × 10^2^ kJ mol^−1^ for CA-20-H-298 (Table S3). At the same time, the polar term becomes strongly negative, indicating that solvation of the ionized species partially compensates for the electrostatic penalty. Because the large positive elec term at pH 7.4 is largely compensated by the oppositely signed polar term, the weaker binding under basic conditions cannot be attributed mainly to the residual electrostatic penalty. Instead, the deprotonated AAc-containing nanoparticles are more highly hydrated and less able to maintain tight nonpolar contact with IBU, so the vdW and nonpolar contributions become markedly less favorable than at pH 2.75. Consequently, the total binding free energies of the AAc-containing systems are generally less negative at pH 7.4 than at pH 2.75 (Figure 3c,d), indicating weaker drug–nanoparticle affinity and a greater tendency for IBU dissociation. At pH 7.4, the weakest binding is observed for CA-15-H-310, with a total binding free energy of only −15.555 × 10^2^ kJ mol^−1^ (Table S3), indicating that this system is the most prone to IBU dissociation under weakly basic conditions at 310 K.

The temperature effect, summarized in Figure 3c,d and Table S3, is secondary to pH for the AAc-containing nanoparticles, but it is clearly composition-dependent. At pH 2.75, heating from 298 to 310 K weakens binding in CA-5 and CA-20, whereas CA-15 shows a slight strengthening (from −23.530 to −24.910 × 10^2^ kJ mol^−1^). At pH 7.4, heating weakens binding in CA-5 and CA-15, while CA-20 becomes slightly more favorable. These non-monotonic trends indicate that the temperature effect is composition-dependent in the AAc-containing nanoparticles. Heating may strengthen PNIPAM-driven nonpolar contact, but this effect can be offset by the hydration and charge effects of ionized AAc, leading to different binding-energy changes among the copolymer systems. For PN-0, the enhanced binding at 310 K under acidic conditions agrees with the typical thermoresponsive collapse of PNIPAM, whereas this trend is markedly weakened at pH 7.4, indicating an additional contribution from the stronger hydration of deprotonated IBU.

### 2.4. Mesh Structure and Steric Control of Ibuprofen Transport

To interpret the geometric correlation between mesh structure and IBU dissociation behavior, we analyzed the geometric requirements for IBU migration using two MD-derived descriptors, the pore limiting diameter (PLD) and the maximum pore diameter (MPD). PLD is the diameter of the largest spherical probe that can continuously percolate through the network and therefore represents the narrowest bottleneck along a connected transport pathway. By contrast, MPD is the diameter of the largest spherical probe that can fit anywhere within the pore space and therefore reflects the size of the largest locally accessible pore region. Because the hydrated nanogel network fluctuates dynamically, PLD and MPD were calculated for 20 uniformly sampled frames from the final 10 ns of the equilibrated MD trajectories, and the resulting values are reported in Table 1 as mean ± SD. In this context, PLD and MPD were used as system-level geometric descriptors of mesh accessibility for outward IBU migration. Accordingly, the IBU dissociated fraction should be understood as an ensemble outcome of 50 molecules sampling heterogeneous local pore environments, rather than as a one-to-one consequence of a single pore-size value. When the effective geometric dimensions of a local pore segment satisfy the steric requirements for molecular passage, IBU molecules in that local region are more likely to lose polymer contact and meet the dissociation criterion.

**Figure 4:**
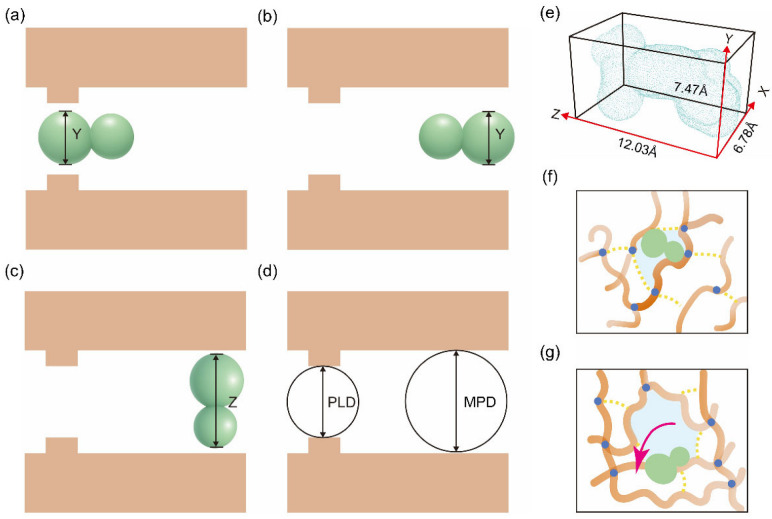
Schematic representation of IBU transport through network pores. (**a**) IBU length along the Y axis is smaller than the PLD. (**b**) IBU length along the Z axis is smaller than the MPD. (**c**) IBU length along the Y axis is smaller than the MPD. (**d**) Illustration of PLD and MPD. (**e**) Three-dimensional size of IBU, with lines and red arrows indicating the measured dimensions along the X, Y, and Z axes. (**f**) Network-restricted IBU transport/dissociation at 298 K and low pH. (**g**) Outward-transport-favored IBU dissociation at 310 K and high pH. In panels (**f**,**g**), the orange network structure represents the crosslinked polymer, the green molecules represent ibuprofen (IBU), the blue dots indicate hydrogen-bonding or crosslinking sites, the yellow dotted lines indicate polymer–polymer hydrogen-bond interactions, and the magenta arrow indicates the outward transport/dissociation direction.

The size match between the solute and the network pores fundamentally influences whether drug diffusion is sterically confined or effectively unconfined. Figure 4e presents the calculated molecular dimensions of the IBU molecule (X = 6.78 Å, Y = 7.47 Å, Z = 12.03 Å). By comparing these dimensions with the PLD and MPD values (Table 1), the dissociation behavior of IBU within the nanopores can be categorized into three representative scenarios.

**Bottleneck-limited state**. When PLD < Y(IBU) = 7.47 Å, the narrowest constriction along the percolating pathway acts as a steric bottleneck. Consequently, IBU molecules located near these pore segments tend to become entrapped within the mesh, resulting in a generally low average dissociated fraction in the system. However, this state does not necessarily imply a zero dissociated fraction, because the cross-linked network does not consist of diffusion pathways with a single characteristic scale. In addition to the bottleneck regions reflected by PLD, the system may contain locally enlarged pore regions reflected by MPD, whose geometric accessibility can be assessed by comparing MPD with the molecular dimensions of IBU (e.g., MPD > Y(IBU), or even MPD > Z(IBU)). Given that 50 IBU molecules are present in the simulation, their initial and evolved distributions may sample either PLD-controlled bottleneck regions or MPD-reflected local free-volume regions. Taking CA-15-H-298 as an example, although PLD = 5.88 Å < Y(IBU), the MPD = 12.46 Å > Z(IBU). Thus, locally enlarged accessible regions may account for the “PLD-confined yet releasable” exception and help explain the variations in dissociated fractions across different systems.

**Transition state**. As shown in Figure 4a,b, when the PLD approaches or slightly exceeds Y(IBU), and the MPD exceeds Y(IBU) but has not yet exceeded Z(IBU), the transport pathway becomes passable but orientation-sensitive. Only specific conformations or orientations allow for easier passage; consequently, the contribution to the overall release enhancement is limited.

**Free state**. When MPD > Z(IBU), the drug molecule becomes less dependent on specific spatial orientations within the accessible regions. In this regime, local steric confinement is markedly reduced; however, substantial outward migration is expected only when polymer–drug affinity is weak, as illustrated in Figure 4c.

The aforementioned classification defines the geometric criteria for the impact of mesh structure on release; however, PLD and MPD fundamentally represent the geometric manifestations of network interactions. The corresponding processes of confinement and deconfinement of IBU within the network pore structure are intuitively visualized in Figure 4f–g. Consequently, the subsequent section proceeds from the perspective of the hydrogen bond network to trace the molecular origins underlying the variations in pore size and drug retention, aiming to achieve predictable regulation of the drug release behavior of nanogels.

### 2.5. Hydrogen-Bond-Mediated Release Mechanism

In the PNIPAM-based nanogels constructed in this study, while chemical cross-links provide the structural framework, reversible hydrogen-bonding interactions serve as a key molecular determinant of both polymer–drug affinity and mesh regulation during IBU release. Two primary categories of hydrogen bonds exist within the system, as quantified in Table 1: (i) polymer–polymer hydrogen bonds, which function as reversible physical cross-linking points to regulate the pore structure; and (ii) polymer–IBU hydrogen bonds, which firmly anchor the drug molecules to the carrier surface while simultaneously competing with polymer–polymer hydrogen bonds for hydrogen bonding sites. As a supplementary assessment of hydration behavior, polymer–water hydrogen bonds were also quantified for all simulated systems (Table S2). In particular, the polymer–water hydrogen-bond statistics in Table S2 show substantially stronger hydration for the deprotonated AAc-containing networks under weakly basic conditions, especially at higher AAc contents. This trend is consistent with the more hydrated environment discussed above, which would weaken polymer–IBU affinity and facilitate outward redistribution of IBU.

This relationship is particularly pronounced under weakly basic conditions at 310 K. The CA-15-H-310 system formed only 10.18 intra-polymer hydrogen bonds, corresponding to a relatively loose network structure, under which IBU exhibited the highest diffusion coefficient and dissociated fraction among all systems. In contrast, the number of intra-polymer hydrogen bonds in PN-0-H-310 was higher (14.61). Although its MPD (12.44 Å) was larger than Z(IBU), its PLD (5.10 Å) was smaller than Y(IBU); consequently, it exhibited only a moderate dissociation tendency. A similar trend was observed when comparing CA-5-H-298 with CA-20-H-298 at the same pH and temperature. These examples suggest that, provided that polymer–drug binding interactions are not significant, a lower number of polymer–polymer hydrogen bonds typically implies larger pore sizes and facilitates diffusion.

Under acidic conditions, the carboxyl groups of both AAc and IBU primarily exist in a protonated state. Consequently, IBU can form extensive hydrogen bonds with the amide groups of PNIPAM and the carboxyl groups of AAc. Table 1 shows that the number of polymer–drug hydrogen bonds in these systems ranges from 4.88 to 9.71. These interactions act synergistically with the intra-polymer hydrogen bonds to effectively retain IBU within the relatively narrow pore channels. Notably, CA-15-L-310 provides strong evidence for the proposed “Affinity Switching” mechanism. Although the CA-15 composition generally exhibits a relatively high dissociation tendency under conditions that are more favorable for dissociation, its IBU dissociated fraction dropped to zero under acidic conditions at 310 K. Together with the relatively high number of polymer–IBU hydrogen bonds and the pronounced enrichment at small r observed for this system, this marked contrast suggests that protonation-induced polymer–drug affinity plays an important role in local drug retention.

Under weakly basic conditions, AAc and IBU were represented in the deprotonated state; consequently, polymer–IBU pairs rarely satisfy the geometric criteria required for hydrogen bonding. Table 1 shows that the number of polymer–drug hydrogen bonds is close to zero in all weakly basic systems, implying that: (i) IBU is no longer directly constrained by local hydrogen bonds, leading to a markedly reduced desorption barrier; and (ii) hydrogen-bonding sites that would otherwise participate in polymer–drug binding become available for intra-polymer rearrangement, thereby facilitating pore-structure adjustment.

Coupled with the thermal motion of IBU at 310 K, this mechanism accounts for why the CA-15-H-310 system exhibits the highest diffusion coefficient and dissociated fraction. In the case of CA-20-H-310, despite the negligible polymer–IBU hydrogen bonding, the release remained low because the high AAc content induced a charge-driven heterogeneous network in which the PLD decreased below the steric threshold required for IBU passage, while residual local van der Waals contacts still contributed to drug retention. Thus, the low dissociated fraction is better attributed to bottleneck-limited transport and local nonpolar retention. Therefore, the regulation of interactions via AAc content, combined with the hydrogen bond network reorganization driven by pH and temperature, constitutes the core mechanism that switches the nanogel from a retention-dominated state to a dissociation-favored state.

To further probe the mechanism suggested by the MD simulations—namely, that polymer–IBU hydrogen bonds directly retain the drug under acidic conditions, whereas polymer–polymer hydrogen bonds intrinsically govern pore size by acting as reversible physical cross-links under weakly basic conditions—we introduced urea as a hydrogen-bond competitor and conducted mechanism-oriented controlled-release experiments. Existing studies have demonstrated that urea can form stable hydrogen bonds with PNIPAM and PAAc, thereby occupying interaction sites on the polymer chains [27,28].

Drawing upon this rationale, urea was added to the release medium in this study. The binding of urea to the polymer produces dual effects: on the one hand, it weakens polymer–polymer hydrogen bonds, thereby reducing the physical cross-linking density and enlarging the pore channels; on the other hand, it competes with IBU for hydrogen bonding sites, directly weakening the polymer–drug hydrogen bonding interactions. If the hydrogen-bond-centric mechanism proposed by MD is valid, the occupation of these hydrogen bonding sites should generally lead to enhanced IBU release.

**Figure 5:**
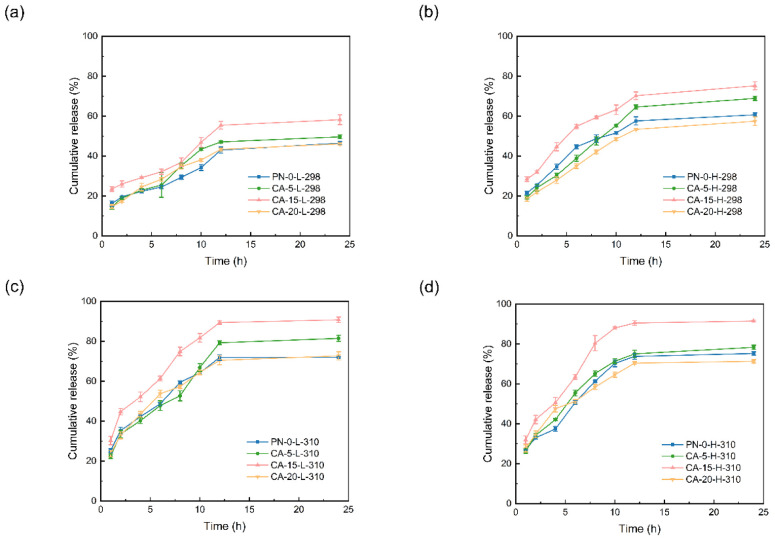
Cumulative IBU release profiles from the nanoparticles in urea-containing release medium under different temperature–pH conditions: (**a**) 298 K, pH 2.75; (**b**) 298 K, pH 7.4; (**c**) 310 K, pH 2.75; (**d**) 310 K, pH 7.4. In each panel, curves correspond to PN-0, CA-5, CA-15, and CA-20 nanoparticles. Cumulative IBU release (%) data are presented as mean ± SD (*n* = 3 independent experiments). Error bars represent SD.

The drug release profiles obtained in urea solutions are consistent with this prediction, as shown in Figure 5a–d. Compared to the urea-free systems, the addition of urea across all 16 combinations of pH, temperature, and composition resulted in a marked increase in the 24 h cumulative release for all systems, with increments generally ranging from 8.9 to 33.0 percentage points. For instance, under acidic conditions at 298 K (Figure 5a), the 24 h release of CA-15-L-298 rose from approximately 37.7% (without urea) to about 58.2%. Even under weakly basic conditions at 310 K, the systems continued to exhibit a release enhancement of approximately 8.9–22.1 percentage points (Figure 5d). This observation is consistent with the MD results for the weakly basic systems at 310 K, which showed negligible polymer–drug hydrogen bonding, suggesting that drug retention was no longer controlled primarily by direct polymer–drug hydrogen bonding, but instead by residual nonpolar interactions together with geometric constraints arising from hydrogen-bond reorganization within the network.

In terms of trends, the release enhancement induced by urea was most pronounced in acidic systems, as illustrated in Figure 5a,c. This is particularly evident for samples such as CA-5-L-298 and CA-15-L-298, which were identified in the simulations as possessing the highest numbers of polymer–drug hydrogen bonds. These systems exhibited strong confinement in the absence of urea; however, upon the occupation of hydrogen bonding sites by urea, the 24 h release quantities rose sharply. This trend supports the view that polymer–drug hydrogen bonds are an important constraint on IBU release.

In weakly basic systems, Figure 5b,d display a corresponding trend. Since polymer–drug hydrogen bonds were already negligible in these cases, urea primarily functioned by weakening polymer–polymer hydrogen bonds to reduce physical cross-linking density and enlarge pore size. Consequently, the release increments were relatively moderate yet substantial.

Together, the urea effects support the dual mechanism suggested by MD: polymer–drug hydrogen bonding is a key determinant of retention under acidic conditions, whereas steric sieving regulated by intra-polymer hydrogen bonding plays an equally important role under weakly basic conditions. Because urea may simultaneously affect hydrogen bonding, polymer hydration/conformation, and ibuprofen solubility in the release medium, the urea-competition experiments are interpreted here as mechanism-supporting perturbation experiments rather than as a fully selective probe of a single interaction type. Accordingly, the stronger release enhancement observed in acidic systems is taken to suggest a larger contribution from disruption of polymer–IBU hydrogen bonding, whereas the more moderate but still clear enhancement in weakly basic systems is consistent with additional contributions from weakening of polymer–polymer hydrogen bonding and mesh opening. These results further support hydrogen-bond-network design as a practical handle for tuning release behavior.

### 2.6. Correlation of MD Descriptors with Experimental Release

A Spearman correlation analysis was performed to evaluate the associations between MD-derived descriptors and the corresponding experimental release behavior across the matched systems [29,30]. To minimize baseline differences among polymer compositions, the correlation was evaluated using within-material centered variables [31].

**Figure 6:**
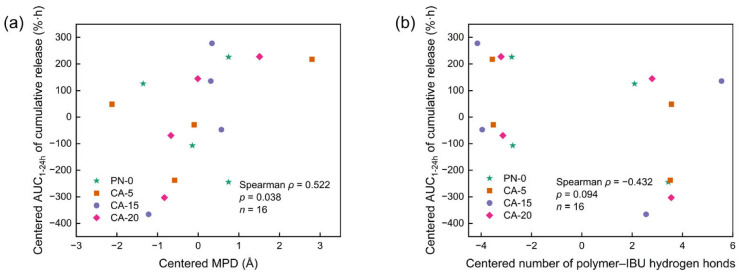
Correlation of simulation-derived descriptors with experimental urea-free release performance. (**a**) Within-material centered Spearman correlation between the MPD and the experimental AUC_1–24h_. (**b**) Within-material centered Spearman correlation between the number of polymer–IBU hydrogen bonds and the experimental AUC_1–24h_.

Among the descriptors examined, MPD showed the clearest monotonic association with the experimental cumulative release performance, quantified by the area under the cumulative release curve from 1 to 24 h (AUC_1–24h_). Specifically, within-material centered Spearman analysis revealed a moderate positive correlation between centered MPD and centered AUC_1–24h_ (*ρ* = 0.522, *p* = 0.038, *n* = 16; Figure 6a). This result indicates that larger locally accessible pore regions are associated with greater overall release extent, consistent with the steric-sieving interpretation developed above.

A complementary trend was observed for polymer–IBU hydrogen bonding. The centered number of polymer–IBU hydrogen bonds showed a moderate negative correlation with centered AUC_1–24h_ (*ρ* = −0.432, *p* = 0.094, *n* = 16; Figure 6b), indicating that stronger polymer–drug hydrogen-bonding interactions tend to suppress overall release. This trend is consistent with the affinity-switching picture discussed in the previous section, in which stronger local polymer–drug interactions favor retention, whereas weakening of these interactions facilitates release.

Taken together, these exploratory correlations provide quantitative trend-level support for the proposed structure–mechanism–performance framework. These descriptors should be interpreted as complementary mechanistic indicators rather than as fully independent regression variables, because several of them are physically coupled and the number of matched systems remains limited.

## 3. Conclusions

In this study, by combining all-atom molecular dynamics simulations with in vitro release experiments, we have elucidated the regulatory mechanisms of pH/temperature dual stimuli and AAc content on the microstructure and IBU release behavior of P(NIPAM-co-AAc) nanogels. The IBU dissociated fraction reflects an ensemble outcome of 50 molecules sampling heterogeneous local pathways within the nanogel, while PLD and MPD collectively describe the overall geometric accessibility of these pathways. Consequently, the CA-15-H-310 system exhibited the highest dissociated fraction.

These findings suggest that the drug release process may not be fully described by the traditional ‘swelling-diffusion’ model alone and can instead be interpreted in terms of two coupled mechanisms: mesh steric sieving (transport determined by bottleneck size) and affinity switching (stimuli-induced variations in polymer–drug interactions). Under acidic conditions, IBU tends to remain enriched within the network, while polymer–drug hydrogen bonds further reinforce local retention and suppress outward transport. Conversely, under weakly basic conditions, enhanced electrostatic repulsion and solvation weaken polymer–drug affinity, while steric sieving associated with the accessible mesh structure remains equally important in governing outward transport. The correlation analysis and MM/PBSA results further support the proposed structure–mechanism–performance framework connecting mesh accessibility and polymer–drug affinity with release behavior.

Certain limitations remain in this study. First, the present atomistic model represents a local cross-linked domain rather than an entire nanogel particle, and only one independently generated network topology was simulated for each composition/pH/temperature condition. In addition, the model does not describe the detailed kinetics of radical polymerization or particle growth. Therefore, the simulations should be interpreted primarily in a comparative and mechanistic sense, rather than as a one-to-one reconstruction of the full ensemble behavior of the nanogel. Second, pH was represented using two limiting protonation-state models for AAc. While this treatment is reasonable for comparing the pH 2.75 and 7.4 conditions studied here, it does not account for partial ionization or local charge-regulation effects in the weak polyelectrolyte network. Further studies are needed to refine the pH treatment and to translate these mechanistic insights into more precise release control.

In summary, this study provides a mechanistic picture of IBU release in P(NIPAM-co-AAc) nanogels under dual pH/temperature stimuli and outlines a quantitative ‘structure–mechanism–performance’ framework. This provides a theoretical foundation and methodological support for the rational design and performance regulation of smart responsive drug delivery systems.

## 4. Materials and Methods

### 4.1. Molecular Dynamics Simulations

#### 4.1.1. Construction of Drug–Carrier Molecular Models

To construct atomistic nanogel models that explicitly account for chemical cross-linking, the model generation was carried out in two sequential stages: (i) network construction by an iterative cross-linking procedure and (ii) all-atom molecular dynamics simulations of the resulting cross-linked nanogels under the target pH/temperature conditions.

In the first stage, pre-polymerized P(NIPAM-co-AAc) copolymer chains and N,N′-methylenebisacrylamide (BIS) cross-linkers were first built in Avogadro 1.2.0 and then packed into a cubic simulation box using PACKMOL 20.15.2 to provide the initial configuration for network formation [32,33]. Each system contained four pre-polymerized linear chains, each with a degree of polymerization of 20 and containing 0, 1, 3, or 4 AAc units (corresponding to 0, 5, 15, and 20 mol% AAc, respectively), together with 50 BIS molecules. The initial packing density was set to 0.8 g cm^−3^ to ensure sufficient proximity between potential reactive sites during the subsequent cross-linking process.

For consistent comparison among the four copolymer compositions, the same network-construction protocol was applied to all systems, with identical numbers of polymer chains and BIS molecules, the same initial packing density, and the same reaction settings. Cross-linking was performed using a self-written cross-linking program based on a template-matching reaction scheme. In each reaction cycle, candidate reactive pairs were first identified according to an intermolecular distance criterion and were then further screened by checking whether the local atomic connectivity matched the predefined pre-reaction template before being converted into the corresponding post-reaction topology [34,35]. The reaction cutoff was progressively increased from 3.5 to 5.0 Å in increments of 0.5 Å, and three reaction searches were performed at each cutoff. After each reaction-search cycle, the updated structure was subjected to a brief relaxation/equilibration step to remove unfavorable local distortions and reduce bond strain before the next cross-linking stage [36]. This iterative procedure was continued until a target reactive-site conversion of 80% was reached or the maximum cutoff was exhausted. After completion of the cross-linking stage, all unreacted BIS molecules were removed, and the resulting BIS-bridged P(NIPAM-co-AAc) networks were retained as the initial nanogel structures for the subsequent molecular dynamics simulations. To enable a controlled comparison across compositions, all systems were generated using the same cross-linking protocol. Such a topology-oriented modeling strategy is consistent with prior MD studies. In cross-linked polymer and hydrogel simulations, stepwise or dynamic bond-formation schemes have been used to generate more realistic cross-linked structures, while representative local network models have also been employed to probe local solute adsorption and interaction environments in swollen polymer networks [37,38,39]. The present atomistic network should be viewed as a representative local cross-linked domain of the nanogel rather than a full-particle model. This model scale was chosen to resolve local mesh accessibility, polymer–drug contacts, and hydrogen-bond reorganization under controlled composition/pH/temperature comparisons while maintaining an explicitly cross-linked all-atom description [39,40].

To represent the pH-dependent environments considered in this work, we adopted two limiting protonation-state models. In the acidic model, all AAc carboxyl groups were assigned as –COOH to represent pH 2.75, whereas in the weakly basic model all AAc carboxyl groups were assigned as –COO^−^ to represent pH 7.4 [41]. This approximation was chosen to compare the two experimentally studied pH regimes within a consistent and computationally tractable atomistic framework. Using a representative pKa value reported for acrylic-acid-based systems (4.75), a simple Henderson–Hasselbalch estimate suggests that the degree of deprotonation is only about 0.99% at pH 2.75 but approximately 99.8% at pH 7.4. Therefore, these two limiting states provide a reasonable approximation for the present acidic and weakly basic conditions [19]. The system temperatures were set to 298 K and 310 K (corresponding to 25 °C and 37 °C, respectively) to represent lower- and higher-temperature conditions relevant to room-temperature and physiological conditions [9]. Because copolymer composition and pH can shift the effective transition temperature, these two temperatures were used here as fixed comparison points across the different systems [13,42]. While it is acknowledged that copolymer composition and pH can shift the specific LCST, fixing the simulations at these two temperatures facilitates a consistent comparison of thermal responsive behaviors across different systems.

To investigate the distribution and interaction of hydrophobic drugs in the vicinity of the P(NIPAM-co-AAc) network, IBU was selected as the model drug, as it is commonly used for PNIPAM-based carriers. The three-dimensional structure of IBU was obtained from the PubChem database [43]. Given the acidic pK_a_ (≈4.9) of IBU [44], IBU was modeled in its deprotonated carboxylate form under weakly basic conditions (pH 7.4), consistent with its dominant charge state in the experimental high-pH medium. To generate a near-network shell initial configuration, the pre-equilibrated P(NIPAM-co-AAc) nanogel was fixed at the center of the simulation box, and 50 IBU molecules were packed by PACKMOL within a spherical shell surrounding the nanogel (i.e., in regions proximal to the nanogel surface rather than throughout the bulk solvent). During packing, a tolerance of 2.0 Å was applied, meaning that atoms belonging to different molecules were initially kept at least 2.0 Å apart to avoid unfavorable overlaps [45].

In this study, the PNIPAM homopolymer is denoted as PN-0, and P(NIPAM-co-AAc) copolymer nanoparticles with AAc contents of 5, 15, and 20 mol% are denoted as CA-5, CA-15, and CA-20, respectively. The system IDs in the matrix sequentially represent “Polymer Type–AAc mole percentage–pH condition–Temperature.” For instance, PN-0-L-298 represents the PNIPAM (PN-0) system at low pH (L) and 298 K, while CA-5-H-310 represents the CA-5 system at high pH (H) and 310 K. A more complete description of the systems, including pH conditions and AAc/IBU states, is detailed in Table S1 in the Supplementary Materials.

#### 4.1.2. Simulation Details

All molecular dynamics simulations were performed using GROMACS 2022.3 [46] with GPU acceleration [47]. The systems were modeled with the SPC/E water model [48], neutralized with counterions, and maintained at a physiological salt concentration of 150 mM NaCl. PBS and HCl were used as experimental media. NaCl was adopted in the simulations as a simplified reference electrolyte. This choice was guided by previous work showing that, for PNIPAM, NaCl captures key features of the PBS response, including the same cloud point temperature and similar post-transition aggregation behavior. Given that NaCl is the dominant salt component in PBS, it serves as a practical and computationally efficient approximation for the major bulk electrostatic screening contribution of the buffer in MD simulations. For the acidic medium, chloride is also present in the HCl solution. Accordingly, NaCl was used here as a reduced common background electrolyte to facilitate controlled comparison between media, although this simplification does not aim to reproduce phosphate-specific ion effects explicitly [49]. The all-atom Generalized Amber Force Field (GAFF) [50] was employed, utilizing the semi-empirical AM1-BCC charge model [51] for atomic charge assignment. Initial topology parameters for the GAFF force field were generated using SOBTOP 1.0(dev5) [52]. GAFF was employed to describe the organic components, and SPC/E was used as the explicit water model. This setup is supported by prior work on related systems: benchmark simulations of aqueous PAA have shown that GAFF-based parametrization can reliably reproduce conformational and hydration properties, indicating that the use of SPC/E together with GAFF is a reasonable choice for acrylic-acid-based components, whereas PNIPAM studies have shown strong water-model sensitivity and have also adopted SPC/E in PNIPAM-based simulations, including reports of experimentally consistent conformational trends and improved reproduction of PNIPAM collapse thermodynamics [53,54].

All simulations followed a four-step protocol: (1) Energy Minimization (EM), (2) NVT equilibration, (3) NPT equilibration, and (4) Production MD runs. For EM, the steepest descent algorithm was employed for a maximum of 50,000 steps using the Verlet cutoff scheme. For NVT equilibration, the system was coupled to the target temperature using the V-rescale thermostat [55] with a coupling time constant of 0.1 ps for 500 ps. In the NPT equilibration phase, isotropic pressure coupling was applied using the Parrinello–Rahman barostat [56] at a target pressure of 1.0 bar with a coupling time constant of 2.0 ps. This phase continued from the NVT stage while maintaining the same temperature coupling parameters. These protocols ensured that the system reached a stable thermodynamic equilibrium prior to the subsequent production runs.

For the production MD runs, temperature coupling was performed using the V-rescale thermostat with a time constant of 0.1 ps, while pressure coupling utilized the Parrinello–Rahman barostat with a time constant of 2.0 ps. The reference temperatures for each system are listed in Table S1, and the reference pressure was set to 1.0 bar. Non-bonded interactions employed the Verlet cutoff scheme, with a cutoff radius of 14 Å applied to both short-range electrostatic and van der Waals interactions. Long-range electrostatics were treated using the Particle Mesh Ewald (PME) method [57,58]. All bond lengths involving hydrogen atoms were constrained using the LINCS algorithm. The equations of motion were integrated using the leap-frog algorithm with a time step of 1.0 fs, and the neighbor list was updated every 10 steps.

The simulation duration for each system was 600 ns; all simulations reached equilibrium, as indicated by the root-mean-square deviation (RMSD) converging to a stable plateau. The RMSD plots for systems at 298 K and 310 K are presented in Figures S1 and S2 in the Supplementary Materials, respectively. Unless otherwise specified, structural and dynamic properties were analyzed using the equilibrated trajectories over the final 10 ns of the production runs. The analyzed properties included diffusion coefficients, radial number density profiles of IBU relative to the polymer center of mass, hydrogen-bond statistics, and interaction energies. Visualization of system configurations and trajectories was performed using Visual Molecular Dynamics (VMD) 1.9.3 [59]. Hydrogen bonds were quantified in VMD using geometric criteria with a donor–acceptor distance cutoff of 3.5 Å and an angle cutoff of 35° [60]. To elucidate the driving forces of network reconfiguration, hydrogen bonds were specifically categorized and statistically analyzed as either intra-polymer (polymer–polymer) or polymer–drug interactions.

The translational diffusion coefficient of IBU was obtained from the mean-squared displacement (MSD) of the center of mass of the drug molecules using the Einstein relation:

$$MSD\left(t\right)={\left\langle \frac{1}{N_{IBU}}\sum_{i=1}^{N_{IBU}}\;{\left|r_{i}\left(t_{0}+t\right)-r_{i}\left(t_{0}\right)\right|}^{2}\right\rangle }_{t_{0}}$$

$$D=\frac{1}{6}\frac{d}{dt}MSD\left(t\right)$$

where $r_{i}$ is the center-of-mass position of the *i*-th IBU molecule, $N_{IBU}$ is the number of IBU molecules, and the angular brackets denote averaging over molecules and multiple time origins. The fitting interval was selected from the equilibrated part of the trajectory where the MSD increased linearly with lag time (590–600 ns). *D* was then calculated from the fitted slope according to $D=\frac{slope}{6}$. The MSD curves of IBU for all simulated systems and the corresponding linear fits used for diffusion-coefficient determination are shown in Figures S4 and S5 in the Supplementary Materials for the 298 K and 310 K conditions, respectively.

#### 4.1.3. Binding Free Energy Calculations

To quantify the interaction strength between the P(NIPAM-co-AAc) carriers and IBU molecules, binding free energies (Δ*G*_bind_) were calculated using the Molecular Mechanics/Poisson–Boltzmann Surface Area (MM/PBSA) method. The calculations were performed using the gmx_MMPBSA v1.6.1 tool [25], which interfaces GROMACS trajectories with AmberTools 20 [26]. Based on the RMSD stability analysis (Figures S1 and S2), 100 snapshots were extracted at equal intervals from the stable trajectory of the last 10 ns of the production runs to ensure statistical reliability.

#### 4.1.4. Geometric Pore Analysis and Molecular Sizing

The pore structure of the cross-linked networks was characterized using PoreBlazer v4.0 [61,62]. The simulation domain was discretized into a cubic lattice, and the Hoshen–Kopelman algorithm was employed to identify percolating pathways. Two geometric descriptors were calculated: the pore limiting diameter (PLD), defined as the diameter of the largest spherical probe that can continuously percolate through the pore network under periodic boundary conditions and thus represents the narrowest bottleneck or window along the connected pathway; and the maximum pore diameter (MPD), defined as the diameter of the largest spherical probe that can be accommodated anywhere within the pore space and thus reflects the size of the largest locally accessible pore region. To account for structural fluctuations in the hydrated network, PLD and MPD were calculated for 20 frames uniformly sampled from the final 10 ns of the equilibrated trajectories, and the resulting values are reported in Table 1 as mean ± SD.

Additionally, to accurately assess the steric compatibility between the drug and the hydrogel mesh, the effective molecular dimensions of IBU were calculated using Multiwfn (version 3.8) [63]. First, the geometry of IBU was optimized at the PBE0/def2-TZVP level using Gaussian 16. Subsequently, quantitative molecular surface analysis was performed in Multiwfn based on the electron density isosurface of 0.0015 a.u., which effectively represents the van der Waals surface of the molecule [64]. The molecular dimensions were determined by measuring the distances between the extrema of the surface vertices along the three principal axes (X, Y, and Z), corresponding to the length, width, and thickness of the molecule, respectively. These dimensions serve as geometric criteria for comparison with the mesh size parameters (PLD and MPD) obtained from MD simulations.

### 4.2. Experimental Section

#### 4.2.1. Materials

Ibuprofen (IBU, AR), N-isopropylacrylamide (NIPAM), and N,N′-methylenebisacrylamide (BIS) were purchased from Shanghai Aladdin Biochemical Technology Co., Ltd. (Shanghai, China). Acrylic acid (AAc), potassium persulfate (KPS), and sodium dodecyl sulfate (SDS) were purchased from Macklin Technology Co., Ltd. (Shanghai, China).

#### 4.2.2. Synthesis of P(NIPAM-co-AAc) Nanoparticles

P(NIPAM-co-AAc) nanoparticles were synthesized via free-radical polymerization with AAc contents of 0, 5, 15, and 20 mol%, respectively. Briefly, 1.8 g of NIPAM, 0.3 g of BIS, 0.05 g of SDS, and the corresponding amount of AAc were dissolved in 160 mL of deionized water. The solution was placed in a three-neck flask equipped with a reflux condenser and stirred continuously. After heating to 80 °C, the mixture was degassed under a nitrogen atmosphere for 30 min. Subsequently, 0.1 g of KPS initiator was added to initiate the reaction, which was carried out under nitrogen at 80 °C for 8 h. The resulting emulsion was centrifuged at 13,000 rpm for 20 min. The product was then transferred into a dialysis bag (molecular weight cutoff: 12,000 Da) and dialyzed at room temperature for 72 h to remove unreacted monomers and other low-molecular-weight impurities. Finally, the nanogels were lyophilized to obtain xerogels for further use.

#### 4.2.3. Ibuprofen Loading and In Vitro Release Assays

IBU was first dissolved in DMSO to obtain a 1 mg/mL solution and sonicated to ensure uniform dispersion. During the polymerization process, the prepared IBU solution was added simultaneously with the KPS initiator; all other procedures remained identical to those used for the synthesis of P(NIPAM-co-AAc).

The in vitro release experiments were performed in 50 mL centrifuge tubes containing 10 mL of release medium. To systematically investigate the release behavior, the media were categorized into two groups: urea-free and urea-containing systems. Specifically, the base media consisted of an HCl solution (adjusted to pH 2.75) and Phosphate-Buffered Saline (PBS, pH 7.4). For the urea-containing group, urea was dissolved in the respective base media at 2.0 M to compete for hydrogen bonds, as this concentration has been reported to be sufficient to significantly perturb the hydrogen-bonding/hydration environment of PNIPAM and alter its phase behavior [65]. The drug-loaded samples were transferred into dialysis bags (molecular weight cut-off: 10,000 Da) and immersed in these solutions. The tubes were then placed in a thermostatic shaking incubator at 25 °C (298 K) or 37 °C (310 K). At predetermined time intervals, 1 mL of the release medium was withdrawn and immediately replenished with 1 mL of fresh medium. The concentration of released IBU was determined by recording UV-vis absorption spectra on a UV-2600 spectrometer (Shimadzu, Kyoto, Japan) at a wavelength of 272 nm. The calibration curve used for quantifying released IBU at different time points is shown in Figure S6 in the Supplementary Materials.

The cumulative drug-release percentage was calculated using the following equation:

$$Cumulative\;release\;(\%)=\sum_{t=0}^{t}\;\frac{C_{t}\times V}{M}\times 100\%$$

where $C_{t}$ (mg/mL) is the concentration at time *t*, *V* is the total volume of the release medium (mL), and *M* is the loaded mass of IBU.

All in vitro release experiments were performed in triplicate (n = 3 independent samples), and the data are presented as mean ± standard deviation (SD). The error bars in the release profiles represent SD. For ease of comparison with the simulation systems, the release experiments were assigned system IDs using the same naming logic: “Polymer type–AAc content–pH condition–temperature”. For example, PN-0-L-298 denotes the PN-0 release system at low pH and 298 K, whereas CA-5-H-310 denotes the CA-5 release system at high pH and 310 K. The media consisted of solutions with or without urea at pH 2.75 or 7.4, and the experiments were conducted at 25 °C (298 K) or 37 °C (310 K). More detailed system information is provided in Table S1 in the Supplementary Materials. Batch-to-batch reproducibility of independently synthesized nanogel samples was not separately evaluated in the present study.

#### 4.2.4. Models for Drug Release Kinetics

To quantitatively evaluate the release behavior of IBU, the urea-free release profiles were fitted to the following kinetic models:

$$\text{Zero-order release:}\;\frac{M_{t}}{M_{\infty }}=k_{0}t$$

$$\text{First-order release:}\;\frac{M_{t}}{M_{\infty }}=1-e^{-k_{1}t}$$

$$\text{Higuchi release:}\;\frac{M_{t}}{M_{\infty }}=k_{H}t^{\frac{1}{2}}$$

$$\text{Korsmeyer–Peppas model:}\;\frac{M_{t}}{M_{\infty }}=k_{KP}t^{n}$$

where $\frac{M_{t}}{M_{\infty }}$ is the fractional cumulative release of IBU at time t, and $k_{0}$, $k_{1}$, $k_{H}$, $k_{KP}$, and n are the corresponding kinetic parameters. The goodness of fit was evaluated by the coefficient of determination ($R^{2}$). *n* is the release exponent, which reflects the release mechanism in the Korsmeyer–Peppas model.

#### 4.2.5. Statistical Analysis of Descriptor–Release Correlations

For correlation analysis, the experimental urea-free release performance of each matched system was summarized by the area under the cumulative release curve from 1 to 24 h, denoted as AUC_1–24h_, which was calculated from the mean cumulative release profile using the trapezoidal rule [66,67]:

$${AUC}_{1-24h}=\sum_{i=1}^{m-1}\;\frac{R_{i}+R_{i+1}}{2}\left(t_{i+1}-t_{i}\right)$$

To reduce the influence of baseline differences among polymer compositions, a within-material centering procedure was applied prior to correlation analysis [31]. For each material *j*, centered variables were defined as $x_{ij}^{\prime }=x_{ij}-{\bar{x}}_{j}$ and $y_{ij}^{\prime }=y_{ij}-{\bar{y}}_{j}$, where $x_{ij}$ and $y_{ij}$ denote the simulation-derived descriptor and the corresponding experimental AUC_1–24h_, respectively.

Monotonic associations between centered simulation-derived descriptors and centered experimental AUC_1–24h_ values were evaluated using Spearman rank correlation [29,30]. The main-text analysis focused on MPD and the number of polymer–IBU hydrogen bonds as representative descriptors of mesh accessibility and polymer–drug affinity, respectively.

## Figures and Tables

**Table 1:** Diffusion coefficients of IBU, IBU dissociated fractions, numbers of polymer–polymer and polymer–IBU hydrogen bonds, and pore geometry parameters (PLD and MPD) for different nanoparticles.

Systems ID	Diffusion Coefficient D (10^−7^ cm^2^ s^−1^)	IBU Dissociated Fraction (%)	Number of Polymer–Polymer Hydrogen Bonds	Number of Polymer–IBU Hydrogen Bonds	PLD (Å)	MPD (Å)
PN-0-L-298	4.67	4	9.60	6.22	6.17 ± 0.71	12.44 ± 0.74
PN-0-H-298	5.97	12	16.28	0.04	5.95 ± 0.60	11.55 ± 0.17
PN-0-L-310	1.52	2	9.80	4.88	6.75 ± 0.61	10.34 ± 0.28
PN-0-H-310	6.09	8	14.61	0	5.10 ± 0.70	12.44 ± 0.87
CA-5-L-298	0.92	0	9.29	7.07	5.66 ± 0.16	10.39 ± 0.48
CA-5-H-298	3.28	8	9.70	0.04	8.70 ± 0.88	10.87 ± 0.51
CA-5-L-310	1.45	0	10.87	7.12	5.30 ± 0.42	8.85 ± 0.54
CA-5-H-310	3.83	10	12.26	0	6.55 ± 0.30	13.77 ± 0.95
CA-15-L-298	1.53	0	12.02	6.71	6.33 ± 0.54	10.67 ± 0.48
CA-15-H-298	4.87	12	8.32	0.2	5.88 ± 0.78	12.46 ± 0.55
CA-15-L-310	1.96	0	9.27	9.71	6.99 ± 0.72	12.20 ± 0.76
CA-15-H-310	8.61	22	10.18	0.01	8.37 ± 0.64	12.23 ± 0.23
CA-20-L-298	1.60	0	10.87	6.77	7.31 ± 0.67	10.51 ± 1.09
CA-20-H-298	1.60	2	10.38	0.08	5.82 ± 0.51	10.67 ± 0.89
CA-20-L-310	2.42	2	11.35	6.01	5.97 ± 0.53	11.33 ± 0.69
CA-20-H-310	1.32	2	5.82	0.01	5.39 ± 1.02	12.85 ± 0.13

**Table 2:** Kinetic modeling results of ibuprofen release.

Systems ID	Zero-Order		First-Order		Higuchi		Korsmeyer–Peppas Model		
	*R* ^2^	*k*_0_	*R* ^2^	*k*_1_	*R* ^2^	*k*_H_	*R* ^2^	*k*_KP_	*n*
PN-0-L-298	−0.211	0.059	0.912	0.172	0.894	0.236	0.961	0.295	0.360
PN-0-H-298	−0.411	0.060	0.902	0.190	0.847	0.244	0.974	0.318	0.337
PN-0-L-310	−0.394	0.060	0.914	0.192	0.852	0.244	0.990	0.310	0.348
PN-0-H-310	−0.410	0.061	0.922	0.202	0.829	0.249	0.931	0.314	0.312
CA-5-L-298	−0.392	0.060	0.930	0.188	0.868	0.242	0.995	0.300	0.382
CA-5-H-298	−0.290	0.060	0.905	0.183	0.865	0.241	0.967	0.302	0.370
CA-5-L-310	−1.130	0.063	0.928	0.248	0.698	0.260	0.999	0.361	0.289
CA-5-H-310	−0.892	0.061	0.921	0.215	0.794	0.249	0.989	0.324	0.408
CA-15-L-298	−0.609	0.060	0.907	0.200	0.831	0.246	1.000	0.330	0.329
CA-15-H-298	−0.328	0.059	0.889	0.179	0.871	0.239	0.996	0.312	0.347
CA-15-L-310	−0.126	0.059	0.886	0.175	0.864	0.239	0.993	0.293	0.366
CA-15-H-310	−0.736	0.062	0.910	0.224	0.767	0.256	0.995	0.350	0.270
CA-20-L-298	−0.751	0.061	0.901	0.209	0.799	0.249	0.969	0.346	0.295
CA-20-H-298	−0.926	0.062	0.880	0.223	0.750	0.254	0.996	0.370	0.256
CA-20-L-310	−0.516	0.062	0.951	0.211	0.827	0.250	0.998	0.304	0.379
CA-20-H-310	−0.954	0.061	0.910	0.224	0.772	0.252	0.999	0.332	0.393

## Data Availability

The data presented in this study are openly available in the article.

## Supplementary Materials

The following supporting information can be downloaded at: https://www.mdpi.com/article/10.3390/gels12050379/s1, Table S1: Systems Simulated in This Study; Table S2: Number of polymer–water hydrogen bonds in all simulated systems; Table S3: Binding free energies (10^2^ kJ mol^−1^) of each system calculated by the MM/PBSA method; Figure S1: RMSD of IBU at 298 K during the MD simulations; Figure S2: RMSD of IBU at 310 K during the MD simulations; Figure S3: Representative final snapshots of ibuprofen-loaded nanoparticles under different temperature (298 and 310 K) and pH conditions. The nanoparticles are shown in orange representation, and IBU molecules are shown in lime CPK representation; Figure S4: Time evolution of the mean squared displacement (MSD) of IBU in the studied systems at 298 K. The linear region was fitted to determine the diffusion coefficient (*D*); Figure S5: Time evolution of the mean squared displacement (MSD) of IBU in the studied systems at 310 K. The linear region was fitted to determine the diffusion coefficient (*D*); Figure S6: Standard calibration curves and corresponding linear regression equations for ibuprofen (IBU) quantification by UV–vis spectrophotometry at 272 nm under different release conditions: (a) pH 2.75, 25 °C; (b) pH 7.4, 25 °C; (c) pH 2.75, 37 °C; and (d) pH 7.4, 37 °C. These calibration curves were used to determine the concentration of released IBU at different time points during the in vitro release experiments.
