# Supplementary material for: Mechanisms of Ibuprofen Retention and Release in Dual-Responsive P(NIPAM-co-AAc) Nanogels: Coupling of Mesh Sieving and Affinity Switching
Source: Gels. 2026 Apr 30;12(5):379. doi: 10.3390/gels12050379 (PMC13205422; doi:10.3390/gels12050379)
Supplement: Supplementary file 1 [file gels-12-00379-s001.zip › gels-4247522-supplementary.pdf]

Table S1. Systems Simulated in This Study

Systems ID	Nanoparticles ID	Polymer	AAc (mol%)	T (K)	Condition label	pH condition	AAc state	IBU state
PN-0-L-298	PN-0	PNIPAM	0	298	Low-pH	2.75	—	All –COOH
PN-0-H-298	PN-0	PNIPAM	0	298	High-pH	7.40	—	All $–COO^-$
PN-0-L-310	PN-0	PNIPAM	0	310	Low-pH	2.75	—	All –COOH
PN-0-H-310	PN-0	PNIPAM	0	310	High-pH	7.40	—	All $–COO^-$
CA-5-L-298	CA-5	P(NIPAM-co-AAc)	5	298	Low-pH	2.75	All –COOH	All –COOH
CA-5-H-298	CA-5	P(NIPAM-co-AAc)	5	298	High-pH	7.40	All $–COO^-$	All $–COO^-$
CA-5-L-310	CA-5	P(NIPAM-co-AAc)	5	310	Low-pH	2.75	All –COOH	All –COOH
CA-5-H-310	CA-5	P(NIPAM-co-AAc)	5	310	High-pH	7.40	All $–COO^-$	All $–COO^-$
CA-15-L-298	CA-15	P(NIPAM-co-AAc)	15	298	Low-pH	2.75	All –COOH	All –COOH
CA-15-H-298	CA-15	P(NIPAM-co-AAc)	15	298	High-pH	7.40	All $–COO^-$	All $–COO^-$
CA-15-L-310	CA-15	P(NIPAM-co-AAc)	15	310	Low-pH	2.75	All –COOH	All –COOH
CA-15-H-310	CA-15	P(NIPAM-co-AAc)	15	310	High-pH	7.40	All $–COO^-$	All $–COO^-$
CA-20-L-298	CA-20	P(NIPAM-co-AAc)	20	298	Low-pH	2.75	All –COOH	All –COOH
CA-20-H-298	CA-20	P(NIPAM-co-AAc)	20	298	High-pH	7.40	All $–COO^-$	All $–COO^-$
CA-20-L-310	CA-20	P(NIPAM-co-AAc)	20	310	Low-pH	2.75	All –COOH	All –COOH
CA-20-H-310	CA-20	P(NIPAM-co-AAc)	20	310	High-pH	7.40	All $–COO^-$	All $–COO^-$

“—" denotes not applicable.

Table S2. Number of polymer–water hydrogen bonds in all simulated systems.

Systems ID	Number of polymer–water hydrogen bonds
PN-0-L-298	26.69
PN-0-H-298	20.76
PN-0-L-310	24.75
PN-0-H-310	19.50
CA-5-L-298	30.10
CA-5-H-298	46.98
CA-5-L-310	30.95
CA-5-H-310	43.18
CA-15-L-298	30.58
CA-15-H-298	83.58
CA-15-L-310	83.59
CA-15-H-310	79.62
CA-20-L-298	29.54
CA-20-H-298	101.82
CA-20-L-310	28.74
CA-20-H-310	104.87

Table S3. Binding free energies (10^2 kJ mol^{-1}) of each system calculated by the MM/PBSA method.

Systems ID	Energy(10^2 kJ mol-1)						
	ΔG_{ele}	ΔG_{vdW}	$\Delta G_{nonpolar}$	ΔG_{polar}	ΔG_{gas}	ΔG_{solv}	total
PN-0-L-298	-0.297	-17.950	-2.470	0.742	-18.247	-1.728	-19.975
PN-0-H-298	0.173	-18.443	-2.576	0.354	-18.27	-2.222	-20.492
PN-0-L-310	-0.342	-23.861	-3.270	0.971	-24.203	-2.299	-26.501
PN-0-H-310	0.1	-18.059	-2.565	0.410	-17.959	-2.155	-20.114
CA-5-L-298	-0.380	-27.093	-3.827	1.102	-27.473	-2.725	-30.198
CA-5-H-298	21.859	-17.633	-2.496	-21.134	4.226	-23.63	-19.404
CA-5-L-310	-0.356	-25.170	-3.538	1.031	-25.526	-2.507	-28.033
CA-5-H-310	19.245	-14.375	-2.081	-18.616	4.87	-20.697	-15.827
CA-15-L-298	-0.349	-21.176	-2.911	0.906	-21.525	-2.005	-23.53
CA-15-H-298	67.375	-15.940	-2.261	-66.097	51.435	-68.358	-16.923
CA-15-L-310	-0.426	-22.459	-3.061	1.036	-22.885	-2.025	-24.91
CA-15-H-310	62.282	-14.602	-2.100	-61.135	47.68	-63.235	-15.555
CA-20-L-298	-0.472	-22.553	-3.091	1.050	-23.025	-2.041	-25.066
CA-20-H-298	95.643	-16.507	-2.330	-93.947	79.136	-96.277	-17.141
CA-20-L-310	-0.314	-19.878	-2.763	0.861	-20.192	-1.902	-22.094
CA-20-H-310	99.606	-18.723	-2.621	-97.666	80.883	-100.287	-19.404

Here,$\Delta G_{gas} = \Delta G_{ele} + \Delta G_{vdW}$,$\Delta G_{solv} = \Delta G_{polar} + \Delta G_{nonpolar}$, and total = $\Delta G_{gas} + \Delta G_{solv}$.

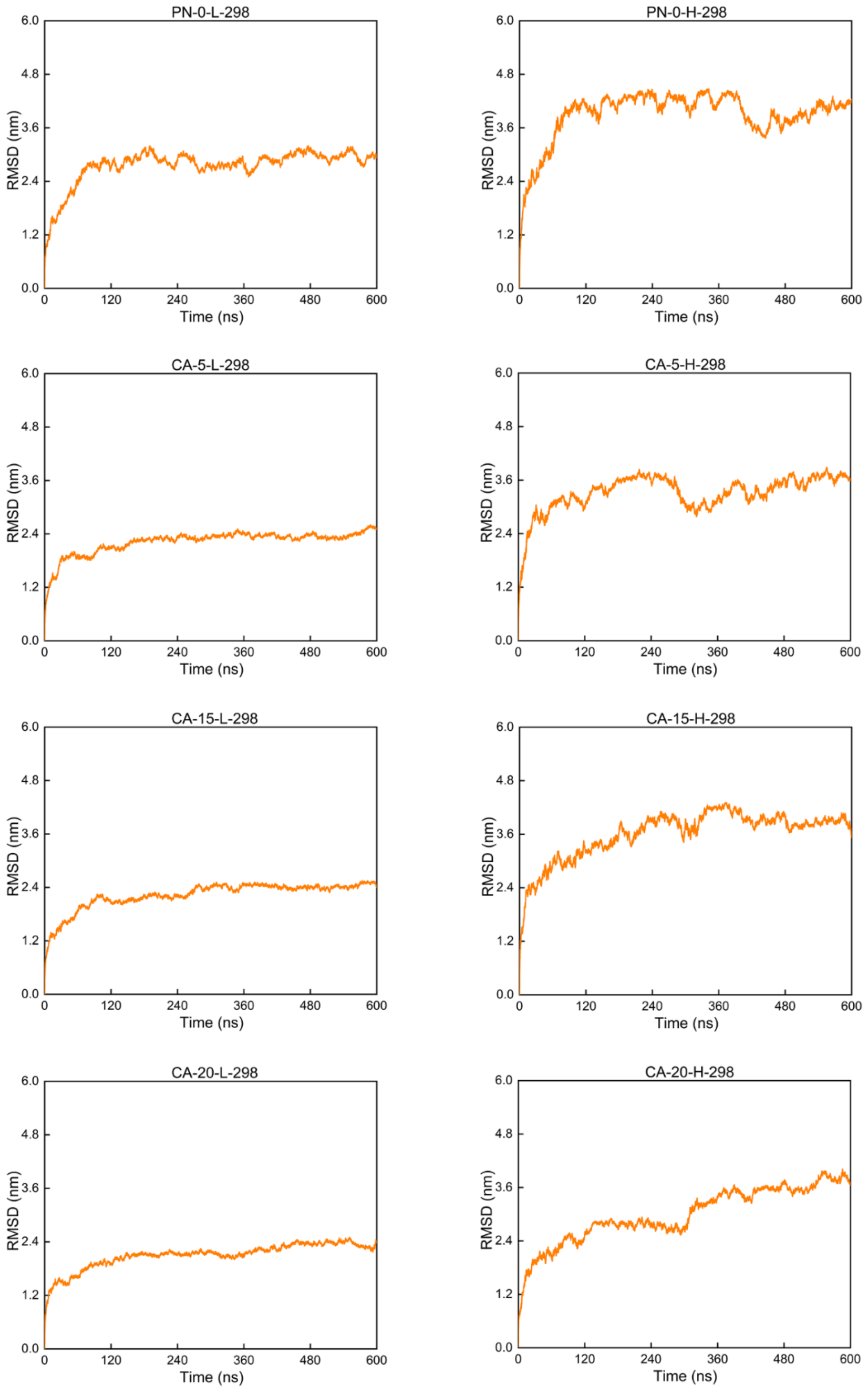

Figure S1. RMSD of IBU at 298 K during the MD simulations.

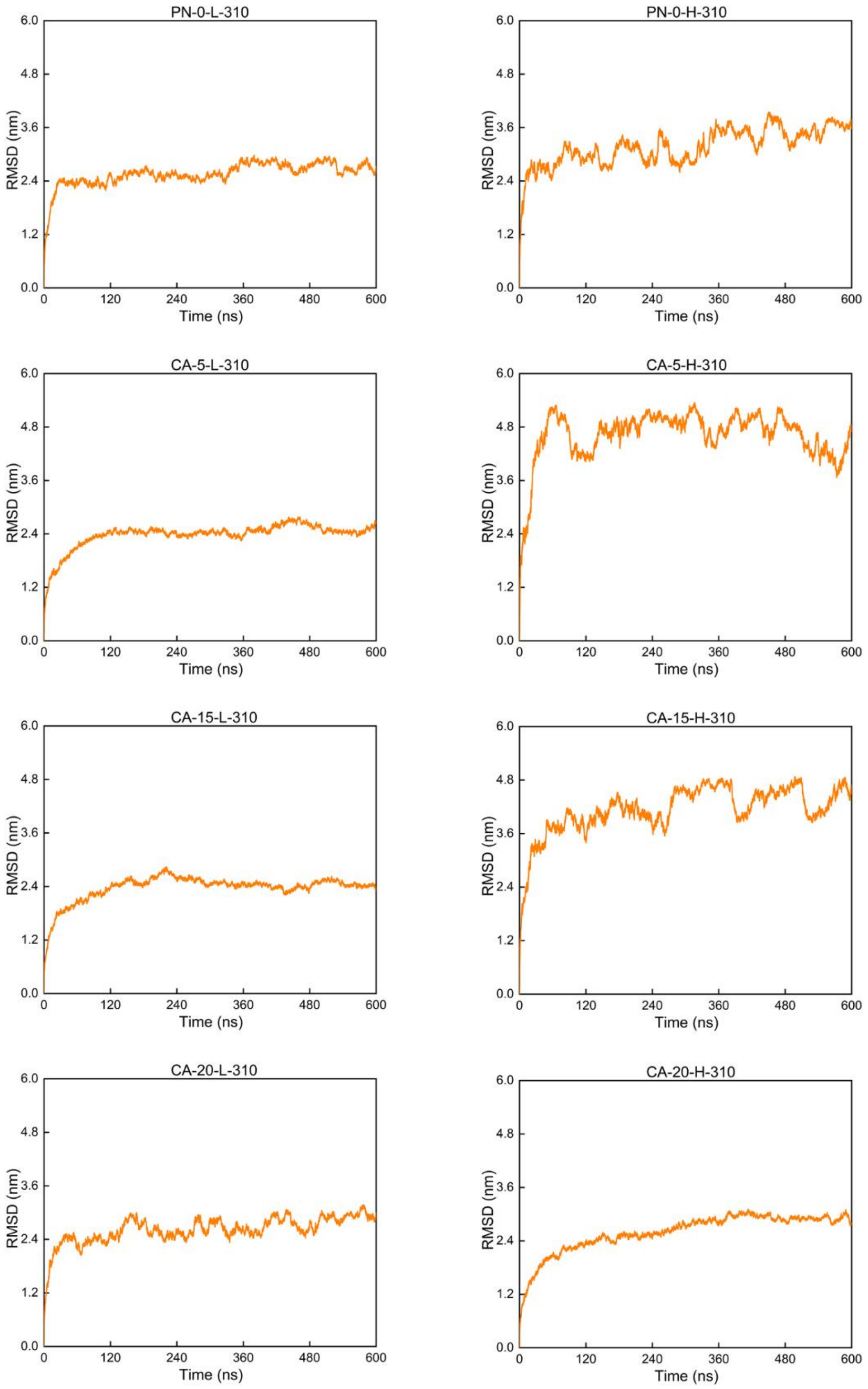

Figure S2. RMSD of IBU at 310 K during the MD simulations.

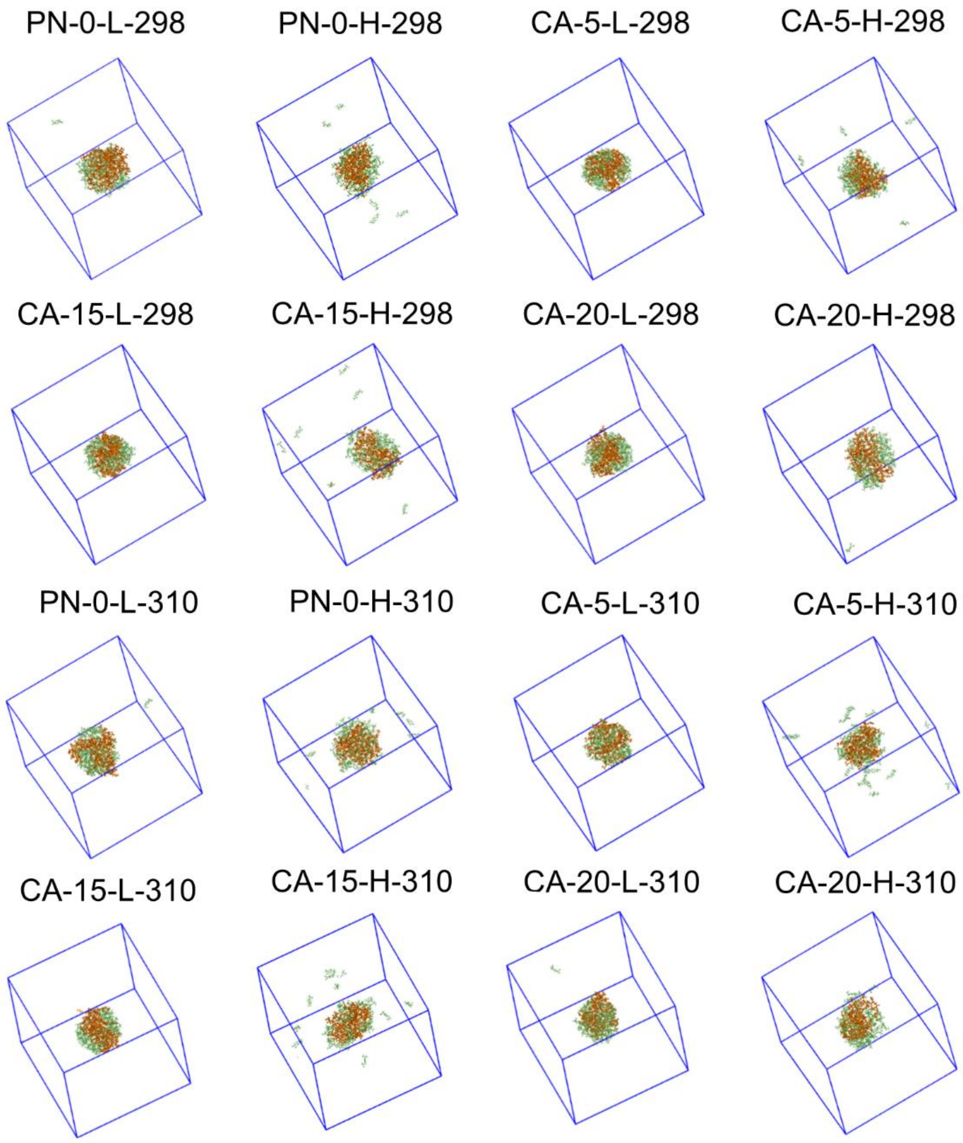

Figure S3. Representative final snapshots of ibuprofen-loaded nanoparticles under different temperature (298 and 310 K) and pH conditions. The nanoparticles are shown in orange representation, and IBU molecules are shown in lime CPK representation.

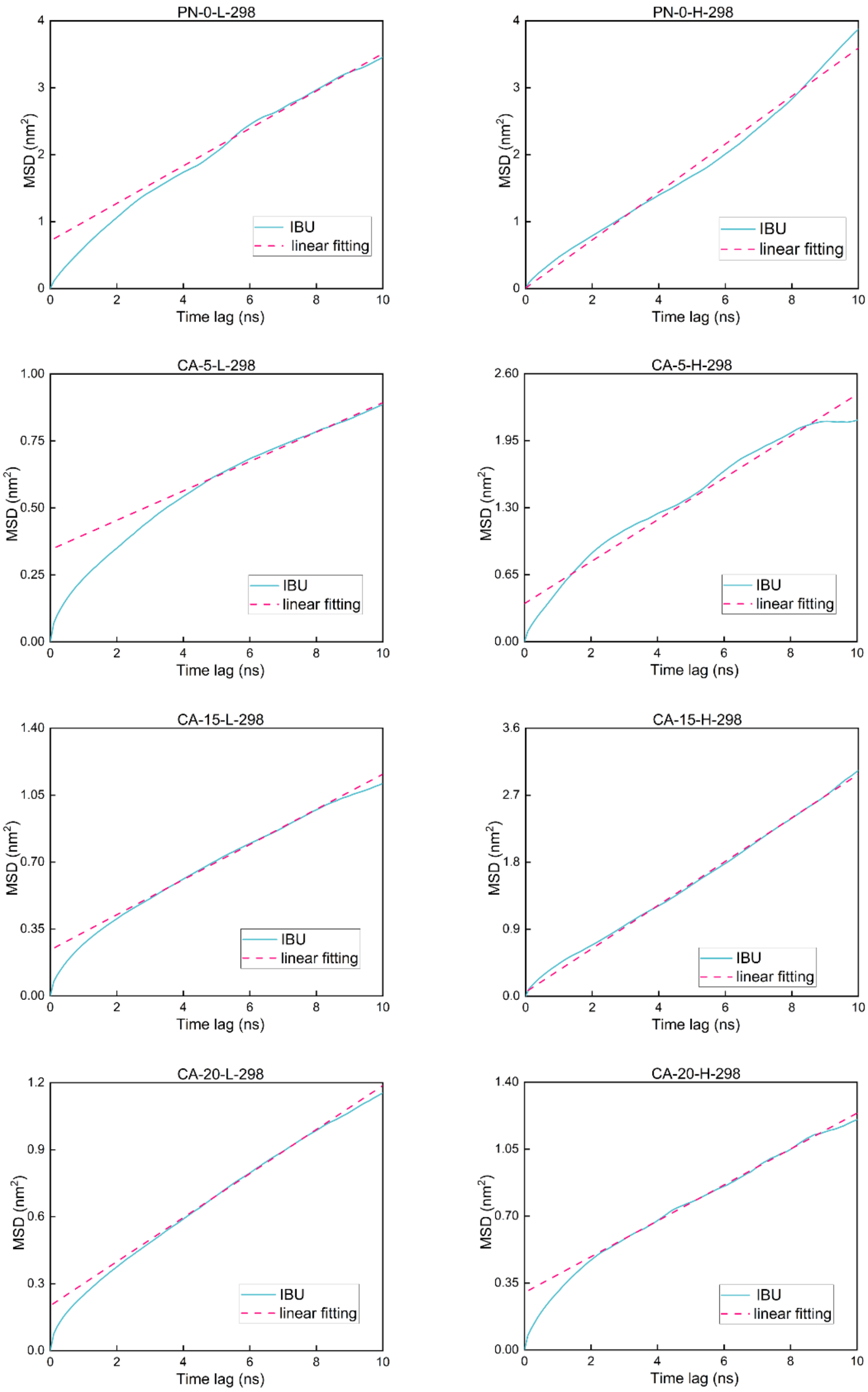

Figure S4. Time evolution of the mean squared displacement (MSD) of IBU in the studied systems at 298 K. The linear region was fitted to determine the diffusion coefficient (*D*).

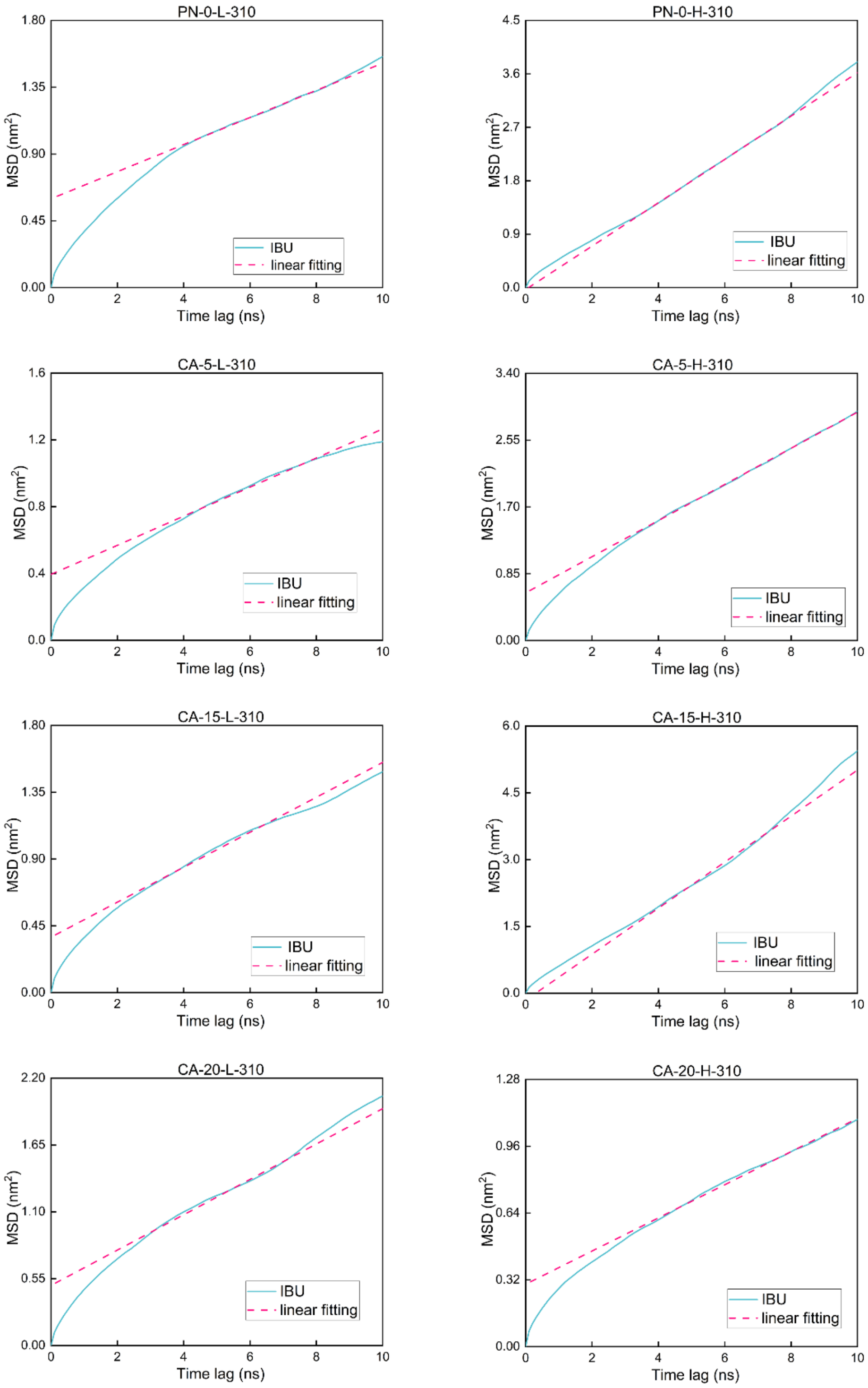

Figure S5. Time evolution of the mean squared displacement (MSD) of IBU in the studied systems at 310 K. The linear region was fitted to determine the diffusion coefficient (*D*).

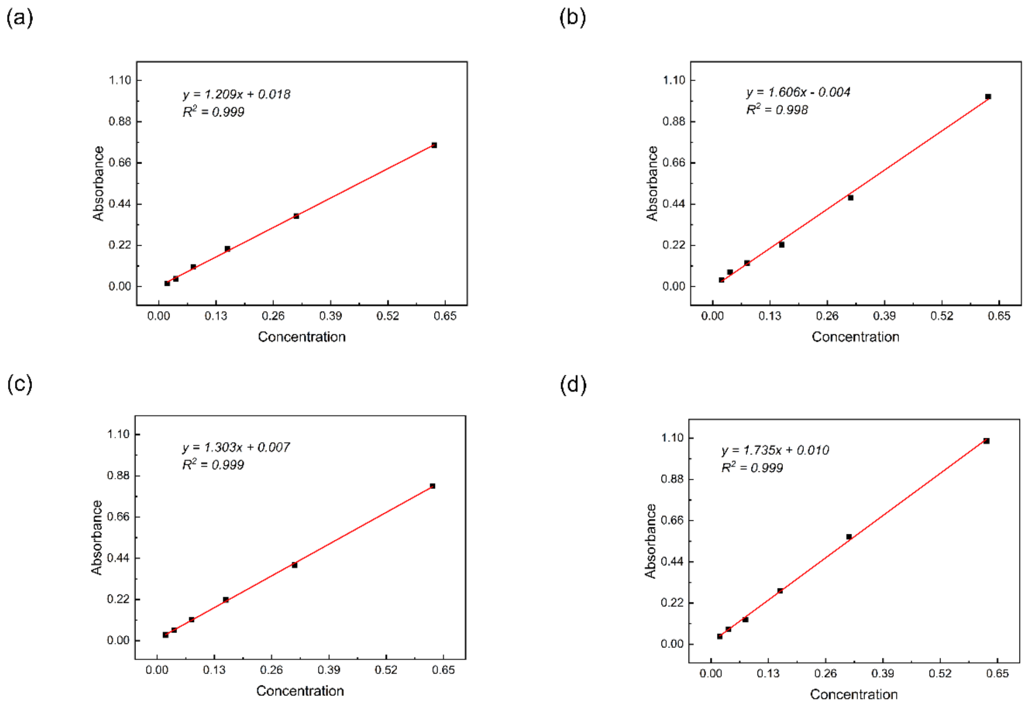

Figure S6. Standard calibration curves and corresponding linear regression equations for ibuprofen (IBU) quantification by UV–vis spectrophotometry at 272 nm under different release conditions: (a) pH 2.75, 25 °C; (b) pH 7.4, 25 °C; (c) pH 2.75, 37 °C; and (d) pH 7.4, 37 °C. These calibration curves were used to determine the concentration of released IBU at different time points during the in vitro release experiments.
